# Hypoxia Effects on *Trypanosoma cruzi* Epimastigotes Proliferation, Differentiation, and Energy Metabolism

**DOI:** 10.3390/pathogens11080897

**Published:** 2022-08-09

**Authors:** Francis M. S. Saraiva, Daniela Cosentino-Gomes, Job D. F. Inacio, Elmo E. Almeida-Amaral, Orlando Louzada-Neto, Ana Rossini, Natália P. Nogueira, José R. Meyer-Fernandes, Marcia C. Paes

**Affiliations:** 1 Trypanosomatids and Vectors Interaction Laboratory, Department of Biochemistry, Roberto Alcantara Gomes Institute of Biology, State University of Rio de Janeiro, Rio de Janeiro 20550-013, Brazil; 2 Institute of Medical Biochemistry Leopoldo De Meis, Center for Health Sciences, Federal University of Rio de Janeiro, Rio de Janeiro 21941-901, Brazil; 3 Tripanosomatide Biochemistry Laboratory, Oswaldo Cruz Institute, Oswaldo Cruz Foundation, Manguinhos, Rio de Janeiro 21040-900, Brazil; 4 Laboratory of Toxicology and Molecular Biology, Department of Biochemistry, IBRAG- UERJ, Rio de Janeiro 20511-010, Brazil; 5 National Institute of Science and Technology—Molecular Entomology (INCT-EM), Brasília 70000-000, Brazil

**Keywords:** hypoxia, ROS, bioenergetics, *Trypanosoma cruzi*, parasite metabolism

## Abstract

*Trypanosoma cruzi*, the causative agent of Chagas disease, faces changes in redox status and nutritional availability during its life cycle. However, the influence of oxygen fluctuation upon the biology of *T. cruzi* is unclear. The present work investigated the response of *T. cruzi* epimastigotes to hypoxia. The parasites showed an adaptation to the hypoxic condition, presenting an increase in proliferation and a reduction in metacyclogenesis. Additionally, parasites cultured in hypoxia produced more reactive oxygen species (ROS) compared to parasites cultured in normoxia. The analyses of the mitochondrial physiology demonstrated that hypoxic condition induced a decrease in both oxidative phosphorylation and mitochondrial membrane potential (ΔΨm) in epimastigotes. In spite of that, ATP levels of parasites cultivated in hypoxia increased. The hypoxic condition also increased the expression of the hexokinase and NADH fumarate reductase genes and reduced NAD(P)H, suggesting that this increase in ATP levels of hypoxia-challenged parasites was a consequence of increased glycolysis and fermentation pathways. Taken together, our results suggest that decreased oxygen levels trigger a shift in the bioenergetic metabolism of *T. cruzi* epimastigotes, favoring ROS production and fermentation to sustain ATP production, allowing the parasite to survive and proliferate in the insect vector.

## 1. Introduction

Chagas disease, which is caused by *Trypanosoma cruzi* parasites, is also known as American Trypanosomiasis because it was traditionally present only in Latin American countries and the southern region of the United States [1]. However, in past decades, it has been increasingly detected in non-endemic areas [2,3]. This illness compromises the work capacity of those infected and can lead to morbidity and death. According to WHO, about 7 million people are infected worldwide and there are 14,000 deaths annually [4]. 

During its life cycle, *T. cruzi* is exposed to different environments with oscillations in temperature, osmotic pressure, pH, and nutritional availability [5,6]. This parasite has shown large plasticity in its energy metabolism along its life cycle. *T. cruzi* epimastigotes preferably catabolize glucose; however, amino acids are an important energy source for epimastigotes in the stationary growth phase [7]. Moreover, metacyclic trypomastigotes decrease glycolytic pathways in relation to epimastigotes [8]. Bloodstream trypomastigotes, on the other hand, seem to rely more on glycolysis rather than oxidative phosphorylation to obtain energy [9]. Alongside amino acids and fatty acids that constitute the main predicted intracellular source of carbon for *T*. *cruzi* amastigotes, glucose also contributes to the energy demands of these proliferative forms [10,11,12].

In eukaryotic cells, energy is largely derived from mitochondria [13]. Mitochondria are a network of organelles that together maintain a variety of cellular functions and processes, such as the level of reactive oxygen species (ROS), cytosolic calcium and apoptosis https://www.ncbi.nlm.nih.gov/pmc/articles/PMC5965678/ R6 [14]. Unlike most eukaryotes, *T. cruzi* has a single mitochondrion extended all around the cellular body. Although mitochondrial energy metabolism may vary along the life cycle of the parasite, *T. cruzi* developmental forms are able to use oxygen to produce ATP during mitochondrial respiration [9,12].

One crucial aspect for cellular metabolism of most eukaryotes is the physiological oxygen flow (normoxia) [15]. Low oxygen levels are also an important aspect of infected and inflamed tissues for reactive oxygen production [16]. In *Leishmania*, evidence demonstrates that oxygen levels of infected tissues are much lower than these currently used in standard cell culture experiments and, in general, correspond to values below 4% O_2_ [17]. In addition to that, hypoxia seems to play a divergent effect on different members of the genus *Leishmania. L. amazonensis*-infected macrophages challenged to O_2_ levels of 5% and 1% resulted in a clearance of the intracellular parasites, an effect not observed in *L. major*-infected macrophages [18,19]. However, there are few studies to date describing how *T. cruzi* developmental forms deal with fluctuations of oxygen levels [20]. 

*T. cruzi* epimastigotes forms inhabit the gut of triatomine insects and have to deal with environmental changes due to blood meal and digestion [5]. Several factors and molecules were shown to be important for the establishment of the parasite in the insect vector, such as the genetic structure of *T. cruzi*, the gut microbiota, and redox status [21,22,23]. However, there are no studies investigating the influence of oxygen levels on parasite infection in their insect vectors. Therefore, the energy metabolism of *T. cruzi* in the face of a hypoxic challenge must be clarified due to a similar environmental condition inside the intestine of the insect. Thus, in the present work we investigated the metabolic adaptation of epimastigotes in response to a hypoxic challenge.

## 2. Results

### 2.1. Hypoxia Induced Proliferation of Epimastigotes, ROS Production, and Inhibition of Metacyclogenesis

**Figure 1:**
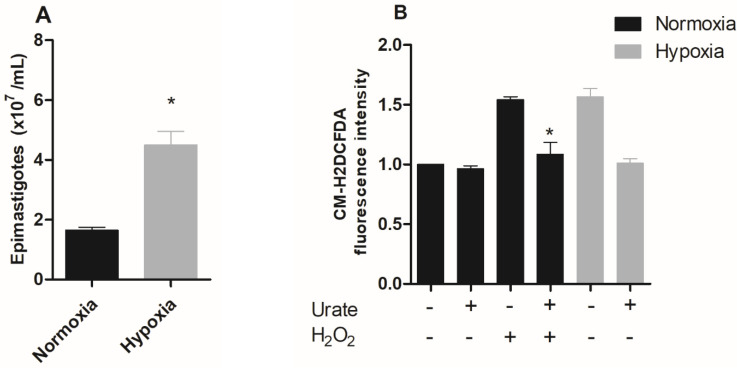

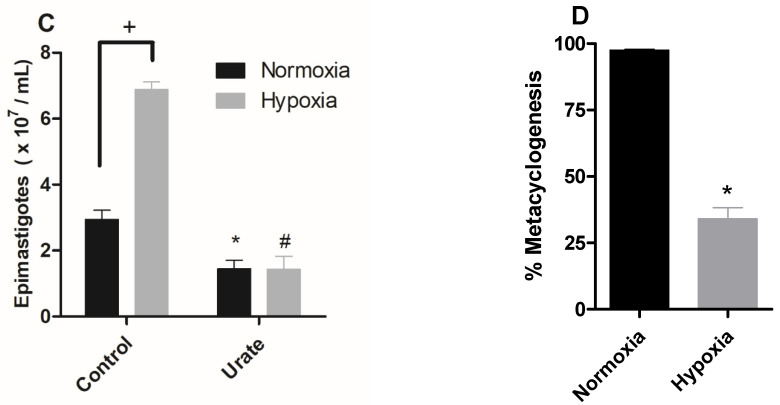
Effect of hypoxic condition on epimastigote proliferation, ROS production, and metacyclogenesis in vitro. (**A**) Epimastigote forms of *Trypanosoma cruzi* (CL Brener) were maintained in normoxia or hypoxia for five days. Parasite proliferation was quantified using a Neubauer chamber. Data are presented as means ± SE of three independent experiments. * *p* < 0.05 in relation to the group cultivated under normoxic conditions by the Student’s *t*-test of variance. (**B**) Epimastigotes (CL Brener) were pre-incubated with 1 mM urate for 15 minutes. Following this incubation, epimastigotes were incubated with 2 µM CM-H2DCFDA in the presence or absence of 100 µM H_2_O_2_. ROS production was quantified by flow cytometry. (**C**) *Trypanosoma cruzi* epimastigotes (CL Brener) in normoxia or hypoxia in the absence (control) or in the presence of 1 mM urate for 5 days. Parasites were quantified in a Neubauer chamber. All data are presented as means ± SE of three independent experiments. * *p* < 0.05 in relation to the control group cultured in normoxia, ^#^ *p* < 0.05 in relation to the control group cultured in hypoxia and + *p* < 0.05 in relation to the control group cultured in normoxia by two-way ANOVA test and post hoc using Bonferroni test. (**D**) *T. cruzi* epimastigote forms (Dm28c) were incubated in TAU 3AAAG in normoxia or hypoxia. After this incubation, the percentage of metacyclic trypomastigotes in the supernatant after 96 h were collected. Results: The data represent means ± standard errors of the percentage of trypomastigotes from three independent experiments, * *p* < 0.05 compared with the control group by the Student’s *t*-test of variance.

Organisms that undergo changes in oxygen levels as a natural event in their life cycle often survive and proliferate under hypoxia. First, we evaluated the effect of hypoxic conditions on epimastigotes of *Trypanosoma cruzi* proliferation and ROS production. The results showed that the epimastigotes maintained in hypoxia for five days proliferated about 2.7 times more than parasites maintained under normoxic conditions (control) (Figure 1A). 

A hypoxic event is accompanied by increased ROS production, especially when the organisms/cells face reoxygenation, an event that forces cells into a major oxidative challenge [24,25,26,27]. Therefore, we characterized the production of reactive oxygen species (ROS) in epimastigote forms subjected to hypoxia for five days after reoxygenation. Hypoxia-cultured parasites showed a 57% increase in ROS production, while the addition of the antioxidant urate reversed this effect to normoxic levels. A very similar profile was observed when epimastigotes were challenged with H_2_O_2_-treated parasites (positive control), which induced a 54% increase in ROS levels. The addition of urate also reversed the increase in the fluorescent signal observed in H_2_O_2_, confirming the specificity of the assay for ROS detection (Figure 1B).

Moreover, ROS production may lead to an increase in proliferation in many cell types [28]. In fact, ROS has already been shown to increase *T. cruzi* epimastigote proliferation [21]. Therefore, we used the antioxidant urate to investigate the involvement of ROS in the proliferation of epimastigotes during hypoxia events. The results demonstrated that urate totally reversed the increase in hypoxia-induced proliferation. Interestingly, parasites maintained in normoxia also demonstrated a reduction in proliferation when treated with urate, thus confirming the participation of ROS in parasite proliferation (Figure 1C).

Since the results demonstrated that hypoxic conditions increased the proliferation of epimastigote forms, we decided to evaluate the effect of the hypoxic condition on the differentiation process, metacyclogenesis. Although clone CL Brener has been used in several studies, the in vitro metacyclogenesis process of this clone is not efficient. Literature data demonstrate that only approximately 20% of the parasites used in the assays differ from the metacyclic trypomastigote form [29]. Therefore, we used clone Dm28c, since the in vitro metacyclogenesis assays in this clone have already been well-described. The metacyclogenesis process was impaired in the hypoxic parasites, showing only 34% of the metacyclic trypomastigotes, while the normoxic parasites showed 97.5% of the metacyclic parasites (Figure 1D).

### 2.2. A Hypoxic Challenge Induces a Remodeling of the Activities of the Mitochondrial Complexes

**Figure 2:**
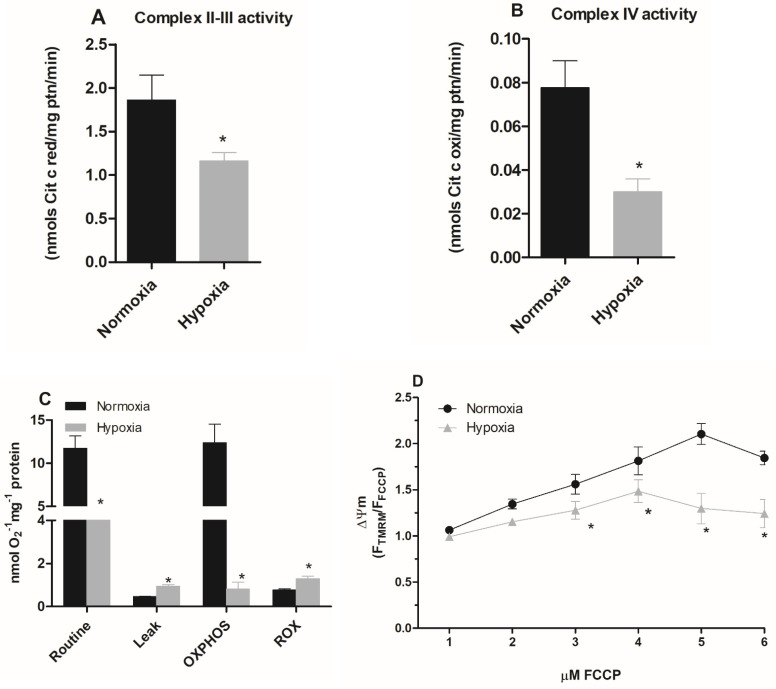
Effect of hypoxia on the activity of epimastigote mitochondrial complexes. *Trypanosoma cruzi* epimastigotes were maintained in normoxia or hypoxia for 5 days. (**A**) The complexes II–III activity was evaluated as the rate of Antimycin A-sensitive ferricytochrome C reduction. The ferricytochrome C reduction was measured by spectrophotometry. (**B**) Complex IV activity was measured as the rate of KCN-sensitive ferrocytochrome C oxidation. * *p* < 0.05 in relation to the normoxic group by Student’s *t*-test. (**C**) Oxygen uptake was quantified on Oxygraph-2K oxygen (OROBOROS Instruments, Innsbruck). The data were corrected for the amount of protein in the samples, and the respiratory rates were calculated. The symbols used * *p* < 0.05 in relation to respiratory control rate of normoxic cells according to Student’s *t*-test of variance. (**D**) Epimastigotes were treated with 50 nM TMRM for 30 min. During the last five minutes, different concentrations of FCCP (1–6 μM) were added. The ΔΨm was calculated using the Fmax/Fmin ratio, where Fmax is the median of normoxic or hypoxic cells treated with TMRM alone and Fmin is the median of normoxic or hypoxic cells treated with a given concentration of FCCP. * *p* < 0.05 in relation to normoxic cells according to the two-way ANOVA test (Bonferroni post hoc test). All data are presented as means ± SE of three independent experiments performed in triplicate.

Various cell types frequently demonstrate the exchange or modification of distinct subunits of the respiratory chain, regulating mitochondrial metabolism, in response to hypoxia [30]. Thus, we evaluated the activity of mitochondrial complexes in parasites maintained under hypoxia. Since the parasite does not have an active Complex I [31], we evaluated the activities of complexes II-III in the epimastigote forms. The results showed that the hypoxic condition reduced the activity of these complexes by 38% (Figure 2A). Additionally, parasites in hypoxia reduced the activity of complex IV by 67% (Figure 2B), supporting the hypothesis that hypoxic events induce mitochondrial remodeling in epimastigote forms.

### 2.3. Parasites Maintained under Hypoxia Show a Reduction in the Mitochondrial Respiration

Since hypoxia impaired the activity of the mitochondrial complexes, we evaluated the oxygen consumption rate of parasites cultivated in normoxia and hypoxia during a reoxygenation event. Parasites maintained in hypoxia for 5 days consumed less oxygen (4.55 ± 0.57 nmol O_2_ · min^−1^ · mg^−1^ protein) than normoxic parasites (11.74 ± 3.21 nmol O_2_ · min^−1^ · mg^−1^ protein) when routine respiration was evaluated. The oxygen consumption after the addition of carboxyatractyloside in normoxia cells was lower (0.43 ± 0.02 nmol O_2_ · min^−1^ · mg^−1^ protein) than in hypoxic cells (0.95 ± 0.14 nmol O_2_ · min^−1^ · mg^−1^ protein), indicating a greater proton leak in epimastigotes maintained in hypoxia. We also assessed the oxidative phosphorylation capacity (OXPHOS), which corresponds to the oxygen consumption values after substrates (succinate + ADP) addition discounted to residual oxygen consumption (ROX). We observed that the OXPHOS of cells under hypoxia (0.98 ± 0.62 nmol O_2_ · min^−1^ · mg^−1^ protein) was much lower than that of cells cultivated in normoxia (12.37 ± 4.34 nmol O_2_ · min^−1^ · mg^−1^ protein). Finally, the evaluation of ROX showed a higher increment for cells maintained in hypoxia (1.3 ± 0.27 nmol O_2_ · min^−1^ · mg^−1^ protein) than those in normoxia. (0.72 ± 0.13 nmol O_2_ · min^−1^ · mg^−1^ protein) (Figure 2C).

We further investigated the mitochondrial membrane potential (ΔΨm) of epimastigotes under hypoxia. We observed that cells cultivated in hypoxia for 5 days presented the highest ΔΨm value at 4 µM FCCP, while for the normoxic cells the highest ΔΨm value was observed at 5 µM FCCP. In addition, the ΔΨm value of cells that remained hypoxic was lower than normoxic parasites after the addition of 3 µM FCCP, showing a 29% reduction in ΔΨm. This effect was also observed at 4 µM, 5 µM, and 6 µM concentrations, with a reduction of 32%, 80%, and 60%, respectively. Therefore, these results demonstrate a reduction in mitochondrial membrane potential in parasites cultured under hypoxia (Figure 2D). 

### 2.4. Hypoxia Induces a Metabolic Shift in Epimastigotes

**Figure 3:**
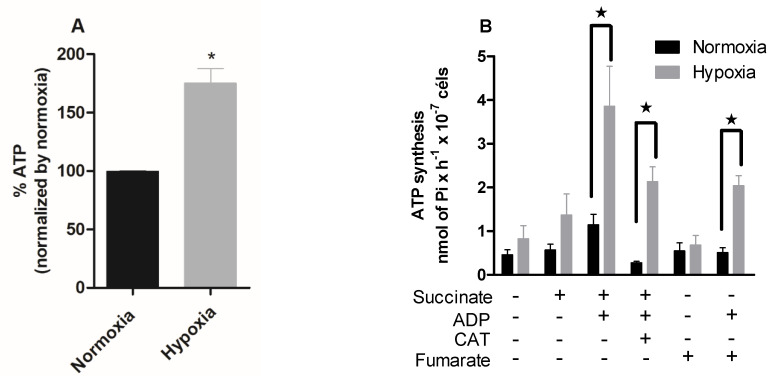
Effect of hypoxia on the intracellular ATP levels in epimastigotes. *Trypanosoma cruzi epimastigotes* were in normoxia or hypoxia for 5 days. (**A**) Intracellular ATP levels were measured using a CellTiter-Glo^®^ Luminescent Cell Viability Assay kit (Promega, Madison, WI, USA). Data are expressed as the percentage of ATP levels relative to the normoxic group, where 100% is equivalent to 0.67 μM ATP. All data are presented as means ± SE of four independent experiments. * *p* < 0.05 in relation to the normoxic group by Student’s *t*-test. (**B**) ATP production was indirectly evaluated by radioactive phosphorylation of glucose, catalyzed by hexokinase (formation of [^32^P] -glucose-6-phosphate). ATP production was measured on a scintillator. All data are presented as means ± SE of three independent experiments. ★ *p* < 0.05 compared to the normoxic group treated with succinate and ADP by the two-way ANOVA test (Bonferroni post hoc test).

We also quantified the intracellular ATP levels of hypoxia-cultured epimastigotes during reoxygenation. The hypoxic condition increased the intracellular ATP concentration by 75% (Figure 3A).

To further investigate the ATP production and concomitant utilization in hypoxia-cultured parasites, we proceeded with a radiometric assay to analyze the ATP synthesis of epimastigotes under different stimuli. In the presence of succinate and ADP, the normoxic parasites showed a 100% increase in ATP synthesis (1.14 nmol of Pi · H^−1^ · 10^−7^ cells). The parasites cultured in hypoxia, when in the presence of these substrates, presented an increase of 182% in ATP synthesis (3.86 nmol of Pi · H^−1^ · 10^−7^ cells). Normoxic parasites in the presence of these substrates and carboxyatractyloside showed a 75% reduction in ATP synthesis. Hypoxic parasites showed a reduction of 44%, being less sensitive to carboxyatractyloside. The *T. cruzi* fermentation process differs from most eukaryotes. The main product released by *T. cruzi* in this process is succinate, which is formed from fumarate. Thus, we evaluated ATP production in the presence of fumarate and ADP, substrates necessary for succinic fermentation, and the normoxic parasites showed no alteration in ATP production. However, parasites cultivated in hypoxia increased ATP synthesis by 200% in the presence of these substrates (Figure 3B).

**Figure 4:**
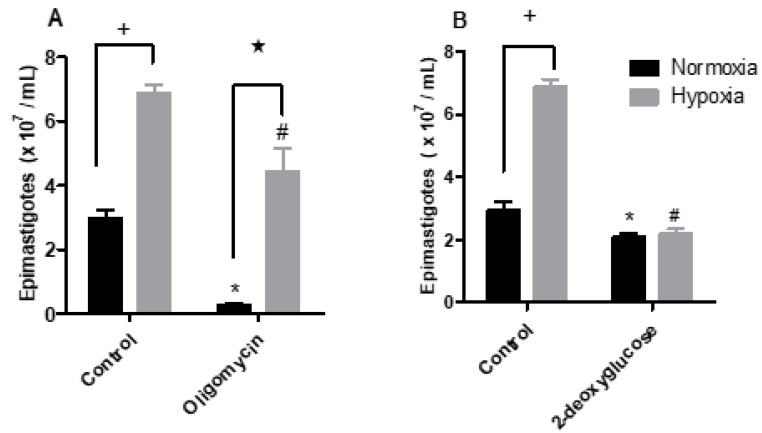
Effect of the metabolic modulators oligomycin or 2-deoxyglucose upon epimastigotes cultured in normoxia or hypoxia. *Trypanosoma cruzi* epimastigotes were maintained in normoxia or hypoxia in the absence (control) and in the presence of (**A**) 2 µg/mL oligomycin or (**B**) 5.5 mM 2-deoxyglucose for 5 days. Parasites were quantified in a Neubauer chamber. All data are presented as means ± SE of three independent experiments. * *p* < 0.05 in relation to the control group cultured in normoxia, ^#^ *p* < 0.05 in relation to the control group cultured in hypoxia and ★ *p* < 0.05 in relation to the control group cultured in normoxia by two-way ANOVA test (Bonferroni post hoc test).

To investigate whether hypoxia-grown parasites produce more ATP through the fermentation process, we performed a proliferation assay using 2-deoxyglucose, which is an inhibitor of hexokinase, the first enzyme of the glycolysis pathway. To evaluate the mitochondrial contribution to ATP supply, we used the ATP synthase inhibitor oligomycin. The proliferation assay showed that normoxic parasites were sensitive to the addition of oligomycin, showing a 91.5% reduction in parasite proliferation. Hypoxic parasites were much less sensitive, with a 35.6% reduction in proliferation (Figure 4A). On the other hand, the addition of 2-deoxyglucose to normoxic cells reduced the proliferation of parasites by 28.8%. The parasites maintained in hypoxia showed a 67.6% reduction in epimastigote proliferation (Figure 4B).

**Figure 5:**
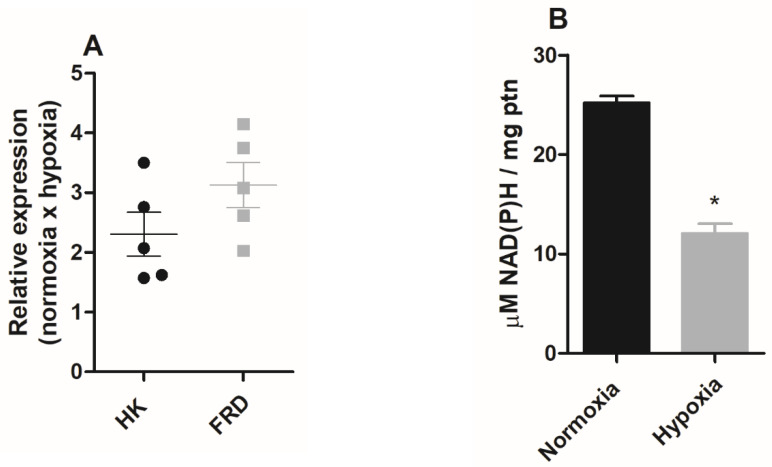
The effect of hypoxia on epimastigote fermentation markers. *Trypanosoma cruzi* epimastigotes were maintained in normoxia or hypoxia for 5 days. (**A**) Hexokinase (*HK*) and NADH-fumarate reductase (*FRD)* gene expression was quantified by RT-qPCR. All quantitative measurements were carried out in triplicate and normalized to the internal *TCZ* (195-bp repeated DNA) control for every reaction. (**B**) Epimastigotes (2 × 10^8^ parasites) were plated in respiration buffer and NAD(P)H was measured by fluorimetry. Results were expressed as mean value ± standard error (SE) of three independent experiments in triplicate. * *p* < 0.05 compared to control according to Student’s *t*-test analysis.

Since in the hypoxia condition ATP production is enhanced and mitochondrial physiology points to the fermentation processes, we evaluated the expression of hexokinase and NADH fumarate reductase enzymes by qPCR in epimastigote forms maintained under normoxic or hypoxic conditions. The assay analyses demonstrated that the hypoxia-grown epimastigote forms exhibited upregulated transcription of both enzymes. Under these conditions, hexokinase increased by 130% and NADH fumarate reductase expression increased by 208% (Figure 5A).

In *T. cruzi*, the fermentation process plays an important role in the oxidation of NADH. However, as the mitochondrial complex I does not participate in oxidative phosphorylation [31], we evaluated NAD(P)H levels in normoxic or hypoxic parasites. The results showed that parasites that remained in culture under hypoxic conditions for five days showed a 52% reduction in NAD(P)H levels (Figure 5B).

## 3. Discussion

Several eukaryotes depend on oxygen supply to proliferate. In fact, it is very common for several cell types to demonstrate stress under hypoxic conditions [32]. On the other hand, cells and organisms that under physiological conditions inhabit hypoxic environments demonstrate an ability to adapt to this context [33,34], and in fact, our results demonstrated that the hypoxic conditions applied here induced the proliferation of epimastigotes and therefore suggest that these parasites are able to adapt to a hypoxic condition. 

Very few studies have addressed a relationship between hypoxia and the parasite, and there are no studies characterizing hypoxia in the insect vector. However, it was demonstrated that the insect vector presents periods of hypoxia alternated with reoxygenation events in response to pH and temperature variation [35]. Furthermore, the microbiota of the hematophagous insect would consume the available oxygen in the insect gut leading to a hypoxic environment [36,37]. Additionally, the microbiota of the insect vector is mostly composed of facultative anaerobic bacteria, and enzymes involved in anaerobic respiration were the major enzymatic determinants in microbiota metagenomic analysis [38,39,40]. This once again suggests that fluctuations of oxygen levels may occur in the triatomine gut. 

Mitochondria consume oxygen during this energy conservation process. Consequently, they are severely affected by lower oxygen availability. The condition of hypoxia alters mitochondrial fusion and fission, mitophagy, and oxidative phosphorylation [30]. The respirometry analysis of epimastigotes cultivated in hypoxia, and then reoxygenated, indicated a decrease in oxygen consumption. Conversely, organisms that do not live in low-oxygen environments increase oxygen consumption during reoxygenation [41,42,43]. The inhibition of oxidative phosphorylation induced by hypoxic challenge is corroborated by the decrease in ΔΨm and in mitochondrial complex activity in epimastigotes. These data justify the low oligomycin sensitivity observed in hypoxia-maintained epimastigotes and support that hypoxia induces a remodeling in the mitochondrial physiology of epimastigotes reducing mitochondrial energy metabolism. 

Many organisms that inhabit low oxygen level environments present a severe reduction of metabolic rates during oxygen deprivation in association with ATP production via fermentative pathways [44]. This phenomenon is recurrently observed in bacteria, fungi, helminths, and even viruses, in which the hypoxic condition induces metabolic changes that lead these parasites to proliferation [45,46,47,48]. However, the hypoxic condition does not reduce *T. cruzi* epimastigotes metabolism, and parasites maintained in hypoxia displayed a significant increase in ATP levels. This might be a consequence of hypoxia-increased glycolytic metabolism, once hypoxia increased expression of glycolytic enzymes such as hexokinase in epimastigotes. In addition, these parasites are much more sensitive to the hexokinase inhibitor 2-deoxyglucose than parasites cultured in normoxia. Thus, these results suggest that increased glycolysis is an adaptive mechanism of the parasite to the hypoxic condition.

During the fermentation process, an increase in reductive equivalents, such as NADH and NAD(P)H levels in organisms that use fermentation, is often observed [49,50]. Epimastigote forms cultivated in hypoxia showed a reduction in NAD(P)H levels. However, specifically, in trypanosomiasis, this may indicate an increase in fermentation as these parasites oxidize NADH in two reactions during the fermentation process [11]. The main end product of fermentation in *T. cruzi* is succinate, which is produced through the reduction of fumarate by the enzyme NADH-fumarate reductase (FRD) [51]. This enzyme participates in the pathway that produces succinate from phosphoenolpyruvate, very common in helminths that inhabit the vertebrate gut, a region that is low in oxygen concentrations [52]. Since parasites maintained under a lower O_2_ pressure showed an increased FRD expression, the fermentation process would be a compensatory mechanism for ATP production in hypoxia-grown epimastigotes. 

The master regulators of the hypoxia response pathway are hypoxia-inducible factors (HIFs). HIFs control a broad range of mechanisms that have key roles in the growth, differentiation, survival, and metabolic activity of cells [53,54]. Hypoxia signaling can act by generating contrasting responses in cells and tissues [55]. Interestingly, HIFs genes were not found in the genome of trypanosomatids [56,57,58]. Additionally, there is still debate about host HIF-1α role during Leishmania infection. HIF-1α expression in mononuclear phagocytes favors the development of visceral leishmaniasis in the spleen and bone marrow [59], while HIF-1α in myeloid cells decreases L. major and cutaneous [60]. On the other hand, studies suggest that Trypanosoma brucei induces HIF-1α degradation in infected macrophages, inhibiting the host cell glycolytic metabolism [61].

Hypoxic cells have been shown to increase the production of reactive oxygen species either along with a hypoxic event or in the reoxygenation process [62]. Parasites in hypoxia increased the production of reactive oxygen species during the reoxygenation process. Moreover, the addition of the antioxidant urate to hypoxia-grown parasites reversed the increase in hypoxia-induced proliferation. These results, therefore, show the production of ROS during the hypoxic event and possibly its participation in the proliferation of these parasites.

Redox status is an important key in the T. cruzi cell cycle. Epimastigotes proliferation is induced by oxidant molecules while antioxidants stimulate metacyclogenesis [21]. The metacyclogenesis of parasites in hypoxia was greatly impaired. It reinforces that the ROS production during the hypoxic event supports epimastigotes proliferation. These data also support that the parasites have the metabolic flexibility to the hypoxic event and subsequent reoxygenation if they occur in the host.

## 4. Conclusions

Therefore, our data set suggests that *T. cruzi* epimastigote forms may adapt to a hypoxic challenge through mechanisms that lead to mitochondrial physiology remodeling and the use of fermentative processes to support the production of ATP required for parasite proliferation. The profile presented by *T. cruzi* suggests that, if there are fluctuations in oxygen levels in the gut of the insect vector, during its biological cycle, the parasites can support this phenomenon.

## 5. Material and Methods

### 5.1. Parasites

The experiments were performed with epimastigote forms of *T. cruzi* (CL Brener) provided by the Trypanosomatid Collection of the Oswaldo Cruz Institute, Fiocruz, Brazil unless otherwise stated. Epimastigotes were maintained at 28 °C for 5 days in Brain Heart Infusion (BHI BD Bacto, Long Beach, CA, USA) medium supplemented with 10% (*w*/*v*) fetal bovine serum (FBS, Vitrocell, Campinas, Brazil) and 30 µM heme (Frontier Scientific, Logan, UT, USA). The medium was changed weekly, and epimastigotes were harvested during the exponential growth phase (4- or 5-day old cultures). Parasite growth was monitored by cell counting in a Neubauer chamber.

### 5.2. Hypoxic Condition

To achieve hypoxia, a pre-analyzed air mixture (95% N_2_/5%CO_2_; Praxair, Danbury, CT, USA) was infused into an air chamber (Billups-Rothenberg, San Diego, CA, USA) constructed with inflow and outflow valves at a flow rate of 20 L/min for 15 min according to the manufacturer’s instructions. The chamber was then sealed and incubated at 28 °C for five days. 

### 5.3. Susceptibility of T. cruzi to Metabolic Modulators 

Epimastigotes (2.5 × 10^6^ parasites/mL) were grown at 28 °C under normoxic (physiological culture conditions) or hypoxic conditions in BHI with 10% FBS in the absence or presence of 1 mM urate (Sigma-Aldrich (St. Louis, MO, USA), 2 µg/mL oligomycin (Sigma-Aldrich, St. Louis, MO, USA), or 5.5 mM deoxyglucose (Sigma-Aldrich, St. Louis, MO, USA), for five days. Afterwards, the parasite proliferation was monitored by cell counting in a Neubauer chamber.

### 5.4. Oxidant Species Production

Epimastigotes (1 × 10^7^ parasites/mL) cultured in normoxia or hypoxia were pre-incubated with urate for 15 minutes. Then, parasites were washed and loaded in PBS (100 mM phosphate buffer and 150 mM NaCl, pH 7.4) with 2 µM CM-H_2_DCFDA (Thermo Fisher Scientific, Waltham, MA, USA) for 30 min. Hydrogen peroxide 100 µM was used as a positive control. The production of the oxidant species was assayed by flow cytometry using a Gallios apparatus with a 488 nm ion-argon laser (Beckman Coulter Inc., Brea, CA, USA).

### 5.5. In Vitro Metacyclogenesis

Differentiation analysis of the metacyclic trypomastigote forms was performed using the *T. cruzi* epimastigote forms of clone Dm28c. The parasites (5 × 10^8^ cells/ml) were washed and maintained for two hours in TAU medium (containing 190 mM NaCl, 17 mM KCl, 2 mM MgCl_2_, 2 mM CaCl_2_, and 8 mM phosphate buffer, pH 6.0) under normal conditions or hypoxia at 28 °C. Then, samples were diluted 100-fold in TAU 3AAAG (TAU supplemented with 10 mM L-proline, 50 mM sodium L-glutamate, 2 mM sodium L-aspartate, and 10 mM D-glucose). The parasites were stored in a 75 cm² cell culture bottle and remained under these conditions for 96 h [63]. Then, 300 µL of the supernatant was collected and stained with rapid panotic (Laborclin Ltda, Pinhais, Brazil) according to the manufacturer’s instructions. Differential counting was performed using the position of the nucleus and kinetoplast as a reference to classify the trypomastigote forms.

### 5.6. Mitochondrial Complex Activities

Mitochondrial complex activities were measured in triplicate at room temperature in a total reaction volume of 1 mL using a spectrophotometer (UV 2550 Shimadzu Co., Shimadzu, Japan). The AA-sensitive succinate:cytochrome c oxidoreductase activity (complex II–III) was measured by the increase in the absorbance at 550 nm due to the reduction of ferricytochrome c [64]. The reaction mixture consisted of 25 mM potassium phosphate buffer, 5 mM MgCl_2_ (pH 7.4), 50 μM horse heart cytochrome c, 10 mM succinate, as an electron donor, and 1 mM KCN, to avoid interference of the IV complex, in the presence of 100 μg of frozen-thawed parasite homogenates. At the end of the reaction, 2 μg/mL Antimycin A was added to evaluate the specificity of ferricytochrome III reduction. The KCN-sensitive cytochrome c oxidase (complex IV) activity was measured based on the decrease in absorbance due to the oxidation of ferrocytochrome c at 550 nm. The reaction mixture consisted of 25 mM potassium phosphate buffer (pH 7.4) and 50 μM sodium dithionite reduced cytochrome c. Decreases in absorbance were monitored after the addition of frozen-thawed parasite homogenates (100 μg of protein). Protein concentrations were determined by the Lowry method [65], using bovine serum albumin as the standard.

### 5.7. Oxygen Consumption Rates

O_2_ consumption rates of the epimastigotes (5 × 10^7^ parasites/chamber) were evaluated by high-resolution respirometry (Oxygraph-2K; OROBOROS Instruments, Innsbruck, Austria) under continuous stirring. The temperature was maintained at 28 °C, and 8.2 µM digitonin was added to permeabilize the parasites in 2 mL of respiration buffer that contained 125 mM sucrose, 65 mM KCl, 2 mM KH_2_PO_4_, 0.5 mM MgCl_2_, 10 mM HEPES-KOH, and 1 mg/mL fat acid-free albumin (pH 7.2) [9]. The rate of oxygen consumption of the epimastigote forms cultured in normoxia or hypoxia was assessed by high-resolution respirometry (Oxygraph-2K OROBOROS Instruments, Innsbruck, Austria). For these measurements, the rate of oxygen consumption of the parasites in their natural physiological state (basal) was measured for fifteen minutes. Subsequently, 10 mM succinate, 4 mM ADP, and 10 µM cytochrome C were added. Leak respiration was stimulated after the addition of 5 µM carboxyatractyloside. Respiration was inhibited by the addition of 2 μg/mL AA to reach residual oxygen consumption (ROX). Routine respiration was calculated by subtracting the ROX value from the basal respiration result. Protein concentration was determined by the Lowry method, using bovine serum albumin as the standard [65]. Digitonin, ADP, oligomycin, carboxyatractyloside (CAT), carbonyl cyanide 4-(trifluoromethoxy) phenylhydrazone (FCCP), antimycin A, succinate, fat acid-free albumin, cytochrome C, KCN, sodium dithionite, and all other reagents of analytical grade were from Sigma-Aldrich (St. Louis, MO, USA).

### 5.8. Mitochondrial Membrane Potential

To detect changes in mitochondrial function, we measured the mitochondrial membrane potential (ΔΨm) using tetramethylrhodamine methyl ester (TMRM, Thermo Fisher Scientific, Waltham, MA, USA). Epimastigotes (1 × 10^7^) were incubated with 50 nM of TMRM for 30 min. During the last five minutes of this incubation, increasing concentrations (1–6 µM) of FCCP were titrated. The F/F_FCCCP_ ratio was used to normalize the results, where F is the mean fluorescence intensity of TMRM (F max) and F_FCCCP_ is the mean fluorescence in the presence of FCCP (F min). Then, the cells were analyzed in a FACSCalibur flow cytometer (Becton Dickinson, Franklin Lakes, NJ, USA), and the fluorescence was detected at 560–590 nm.

### 5.9. ATP Quantification

Intracellular ATP levels were measured by the CellTiterGlo Luminescent Cell Viability Assay (Promega) where the signal is proportional to the ATP concentration. Epimastigote forms cultured in normoxia or hypoxia were washed, and the parasite concentration was adjusted to 1 × 10^7^cells in 200 µL of PBS. Then, 50 μL corresponding to 2.5 × 10^6^ epimastigotes were transferred into opaque-walled 96-well plates, and 50 μL of the CellTiter-Glo reagent were added directly into each well; then, the plates were incubated in the dark for 10 min. The bioluminescence was measured using a GloMax-Multi Microplate Multimode Reader (Promega), and ATP concentrations were calculated from the ATP standard curve.

The net synthesis of ATP was also measured through the incorporation of [^32^P]-Pi into ADP, forming [γ-^32^P]-ATP. Radioactive inorganic phosphate (^32^Pi) was purchased from the Instituto de Pesquisas Energéticas e Nucleares (IPEN, São Paulo, Brazil) as orthophosphoric acid and was purified by extraction in a phosphomolybdate complex with a mixture of 2-butanol/benzene followed by re-extraction into the aqueous phase with ammonium hydroxide and then precipitation as a MgNH_4_PO_4_ complex [66]. The radiolabeled [γ-^32^ P]-ATP was detected by its use as a substrate for the phosphorylation of glucose to glucose-6- ^32^P by hexokinase, which was further extracted and quantified in a scintillation counter, as previously described [67]. Briefly, epimastigote forms (5 × 10^8^ cells/mL) were maintained in normoxia or hypoxia for five days. In this assay, cells were permeabilized with 8.2 µM digitonin and added to the [^32^P] Pi reaction medium, allowing for the evaluation of [γ-^32^P] ATP production and, consequently, [^32^P]-glucose-6-phosphate by the enzyme hexokinase. The quantification of ATP production was measured indirectly, as previously described with minor adaptations [68]. The assay was performed at 28 °C in 500 µL of a reaction medium containing 50 mM HEPES-Tris (pH 7.4), 5 mM 5′AMP, 5 mM MgCl_2_, [^32^P] Pi 4 μCi/μmol, glucose 10 mM, 120 mM NaCl, 30 mM KCL, 25 U hexokinase and 8.2 µM digitonin. Aliquots of 10 mM succinate, 1 mM ADP, 5 μM carboxyatractyloside, and 400 μM fumarate were added to the appropriate treatments. The reaction was initiated by the addition of cells and 1.5 hours later stopped by the addition of 400 µL of 40% ammonium molybdate. Free [^32^P] Pi was removed by three washing procedures using 1 mL 100% acetone and 400 µL 100% butyl acetate. Then, 0.4 mL aliquots of the supernatants were added to a filter paper and the amount of [^32^P] -glucose-6-phosphate was counted in a scintillator (Model Tri-Carb—Perkim Elmer, Waltham, MA, USA).

### 5.10. Differential Expression of Hexokinase and NADH Fumarate Reductase Enzymes

The expressions of the hexokinase and NADH fumarate reductase enzymes were evaluated by qPCR. To perform these analyses, RNA was extracted from the epimastigotes forms by the TRIzol method using the manufacturer’s instructions (Invitrogen, Waltham, MA, USA). cDNA synthesis was performed using the High Capacity cDNA Reverse Transcription Kit (Applied Biosystems, Waltham, MA, USA) according to the manufacturer’s instructions. The qPCR mix was done using a Sybr Green PCR Master Mix (QIAGEN, Hilden, Germany). All quantitative measurements were normalized to *T. cruzi* 195-bp repeated DNA (TCZ) as an internal control for every reaction [69]. Results were expressed as mean value ± standard error (SE). The mRNA fold change was calculated by the following equations: ^Δ^*C_T_*=^Δ^*C_T_*_(target)_−^Δ^*C_T_*_(TCZ)_; ^ΔΔ^*C_T_* = ^Δ^*C_T_*_(hypoxia)_−^Δ^*C_T_*_(normoxia)_; mRNA fold change = 2^−ΔΔ*C*^*_T_* [70]. The primers for the hexokinase enzyme gene (TcCLB.510121.20: esmeraldo-like and TcCLB.508951.20 non-esmeraldo-like, TriTryp_DB) used were: AGATCAGTGCCATGTTCTGC (sense) and ACGGTGTCTTCTCAAACACG (antisense). TCZ-specific primers were TCZ1–5′-CGAGCTCTTGCCCACACGGGTGCT-3′ and TCZ2–5′-CTCCAAGCAGCGGATAGTTCAGG-3. The oligonucleotide primers for the fumarate reductase enzyme gene (TcCLB.503841.80: esmeraldo-like TriTryp_DB) used were: ACGTGAGATGTTGTCGCAGA (sense) and ACGAAGGGTATACGGCACAG (antisense). 

### 5.11. Quantification of Intracellular NAD(P)H Levels

Quantitation of the NAD(P)H levels was performed in epimastigote forms (2 × 10^8^ epimastigotes per replicate) maintained at normoxia or hypoxia for five days. The parasites were then washed twice with respiration buffer, pH 7.2, and quantification was performed by fluorimetry using the autofluorescent property of the NAD(P)H molecule (λ excitation: 337 nm and λ emission: 445 nm) [71]. NAD(P)H concentrations were calculated from a standard NAD(P)H curve performed in the experiment (4–500 µM). The protein content of the samples was quantified by the Lowry method [65], and fluorimetry data were normalized by the protein concentration.

### 5.12. Statistical Analyses

Statistical analyses were performed using Graph Pad Prism, Version 3.0 (Graph Pad Software, Inc., San Diego, CA, USA). Results were expressed as mean ± standard error (SE) of independent experiments or as mean ± standard deviation (SD) of a representative experiment. The Student’s *t*-test, one-way ANOVA, and two-way-ANOVA tests were used. After the last two tests, Tukey (one-way ANOVA) or Bonferroni (two-way-ANOVA) post hoc tests, respectively, were applied to verify if there was a difference between treatments with significance * *p* < 0.05.

## Data Availability

The data is all available for free access.
